# Patience

**Published:** 1887-03-26

**Authors:** Hannah F. White


### PATIENCE.

"For ye have need of patience, that, after ye have done the will of God, ye might receive the promise."—HEB. x, 36.

*Written during a period of long-continued suffering and weakness.*

Give me patience, dearest Father,
Patience in the strife;
Patience underneath the trying
Discipline of life;
Patience founded on the Rock,
Which can stand the rudest shock.

Give me patience, O my Saviour,
Patience to be still,
When some hasty word of others
Contradicts my will;
That my life may ever be,
Worthy of Thy love to me.

Give me patience, O my Father,
When my heart is sore,
When the thorn in love Thou sendest
Presses more and more;
Then O let Thy grace be known,
By Thy strength in weakness shown.

Give me patience, O my Father,
When the hours seem long;
When through weary, restless weakness,
Hushed is praise or song;
Then, O Christ, to Thee I flee,
Thou who bore my pains for me.

Thus, O Father, give me patience
Always to abide,
Ever waiting, ever watching,
Close at Jesus' side:
Till the shining of Thy day
Shall chase the clouds of earth away.

HANNAH F. WHITE.

